# Genetic Polymorphisms of ACE1 Rs4646994 Associated with Lung Cancer in Patients with Pulmonary Nodules: A Case–Control Study

**DOI:** 10.3390/biomedicines11061549

**Published:** 2023-05-26

**Authors:** Rong Qiao, Siyao Sang, Jiajun Teng, Hua Zhong, Hui Li, Baohui Han

**Affiliations:** 1 Shanghai Chest Hospital, Shanghai Jiao Tong University School of Medicine, Shanghai 200030, China; qiaoxkyy@hotmail.com (R.Q.); seven_tjj@sina.com (J.T.); 2 MOE Key Laboratory of Contemporary Anthropology, Human Phenome Institute, School of Life Sciences, Fudan University, Shanghai 200438, China; 20110700057@fudan.edu.cn; 3 Fudan-Datong Institute of Chinese Origin, Datong 037006, China

**Keywords:** genetic polymorphism, ACE1 rs4646994, lung cancer, pulmonary nodules

## Abstract

Background: Currently, many detection methods have high sensitivity to the diagnosis of lung cancer. However, some postoperative patients with pulmonary nodules are eventually diagnosed as having benign nodules. The ideal evaluation of an individual with a pulmonary nodule would expedite therapy for a malignant nodule and minimize testing for those with a benign nodule. Methods: This case–control study is designed to explore the relationship between ACE1 rs4646994 polymorphism and the risk of lung cancer in patients with pulmonary nodules, for which 400 individuals with lung cancer and benign pulmonary nodules were included. A DNA extraction kit was used to extract DNA from peripheral blood. The relationship between ACE1 rs4646994 and the risk of lung cancer in patients with pulmonary nodules was determined by the chi-square test, logistic regression analysis and cross analysis. Results: The results showed that after adjusting for age and gender confounding factors, the risk of lung cancer in patients with pulmonary nodules carrying the DD genotype was more than three times that of the I carriers (II + ID) genotype (OR = 3.035, 95% CI, 1.252–7.356, *p* = 0.014). There was no significant difference between lung squamous cell carcinoma and lung adenocarcinoma in the polymorphism of ACE1 rs4646994 (*p* > 0.05). We also found that the ACE1 rs4646994 DD genotype frequency was inversely correlated with the risk of EGFR mutation in lung adenocarcinoma patients. Conclusions: Our study indicated that ACE1 rs4646994 polymorphism increases the risk of lung cancer in patients with pulmonary nodules from China.

## 1. Introduction

Lung cancer is a primary cancer type with high incidence and mortality, and the number of new cases of lung cancer in China has continued to rise [1]. The overall 5-year survival rate of lung cancer patients is less than 15%, but the 5-year survival rate of early lung cancer can be increased to more than 80% after reasonable treatment [2]. Lung cancer is classified as small cell lung cancer (SCLC) (15% of total diagnoses) and non-small cell lung cancer (NSCLC) (85% of total diagnoses) [3]. In the classification of NSCLC, adenocarcinoma (LUAD) is the most common subtype of lung cancer, followed by squamous cell carcinoma (SCC) [4].

A pulmonary nodule is an abnormal area in the lung that is less than 3 cm in size, and most pulmonary nodules are benign (not caused by cancer) [5]. The risk of cancer increases with pulmonary nodules that are larger in size. Risk factors for increasing the likelihood of lung nodule carcinogenesis include the following: current or previous smoking, older, personal cancer history, family history of lung cancer, emphysema, exposure to asbestos or radon and EGFR mutation [5,6,7,8]. This suggests that the development of pulmonary nodules into lung cancer is a complex and gradual process [9]. In recent years, several studies have reported the correlation between single nucleotide polymorphisms (SNPs) of genes related to metabolism, DNA damage repair, cell cycle regulation and lung diseases [9,10,11,12]. The treatment of patients with pulmonary nodules should be guided by the probability of malignant nodules, the safety of detection, and other additional tests [13].

Angiotensin-converting enzyme (ACE) encodes an enzyme involved in blood pressure regulation and electrolyte balance that converts angiotensin I into a physiologically active peptide angiotensin II, and degrades bradykinin [14]. ACE plasma levels depend on the 287 bp insertion/deletion (I/D) polymorphism of the ACE gene on chromosome 17q23 [15]. According to its variation, there are three different genotypes: II, ID and DD [15]. It has been reported that the D allele is associated with increased ACE expression, and it has been observed that D allele heterozygous carriers have higher levels of systemic ACE protein than I allele carriers [16,17,18]. A large number of studies have reported that ACE1 rs4646994 polymorphism is associated with a variety of diseases, including cardiovascular disease, psoriasis, kidney disease, stroke and Alzheimer’s disease [14,19,20]. A few studies have reported that ACE1 rs4646994 polymorphism may be a possible risk factor for lung cancer [12]. Sergej Nadalin et al. showed that ACE-I/D polymorphism increased the risk of nicotine dependence and smoking severity in lung cancer patients in a gender-specific manner [9]. On the basis of previous relevant studies, the aim of this study was to investigate the possible correlation between ACE1 rs4646994 polymorphism and lung cancer in patients with pulmonary nodules, providing evidence for molecular markers and the etiological study of lung cancer.

## 2. Materials and Methods

### 2.1. Ethical Conformity

This pilot study was approved by the Research Ethics Committees of the Shanghai Jiao Tong University, under protocol KS1407, in the city of Shanghai, China in 2014. All participants signed their informed consent.

### 2.2. Case and Control

For our study, IRB approval and a waiver of written informed consent were obtained. Inclusion criteria are as follows: (1) Patients must be greater than or equal to 18 years old, regardless of gender. (2) Patients have positive pulmonary nodules found by conventional dose CT or low-dose spiral CT (LDCT): (a) non-calcified nodules with a diameter of 5–20 mm; and (b) all three imaging types of pulmonary nodules can be included in the group, namely solid nodules, partial solid nodules (mixed ground glass density nodules) and pure ground glass density nodules. (3) Patients are prepared to receive surgical treatment of pulmonary nodules. (4) Patients have newly diagnosed pulmonary nodules who received baseline CT within 60 days before enrollment. (5) Patients must be able to fully understand the informed consent, agree to participate in the study and sign the informed consent. The exclusion criteria are as follows: (1) pregnant or lactating women; (2) patients received any diagnostic puncture treatment before enrolled the group, such as percutaneous lung biopsy, bronchoscopy biopsy or surgery; (3) receive any blood transfusion within 30 days before enrollment; (4) patients with any malignant tumor (except for non-melanoma skin cancer) diagnosed by pathology 2 years before enrollment; and (5) unable to understand or sign informed consent. Finally, subjects with lung cancer (n = 300) were included as the case group and matched with subjects with benign nodules (n = 100) as the control group. Both groups were investigated for clinical demographic data, including age, gender, and histological type.

### 2.3. Genotyping

TaqMan^®^-MGB probe assays were used to genotype the polymorphisms in 384-well plates on LightCycler^®^ 480 system (Roche Ltd., Basel, Switzerland). The primers and probes of TaqMan^®^ assays were designed using Primer Express Oligo Design software v3.0 (Applied Biosystems, Foster City, CA, USA). PCR reactions were performed in a 6 μL reaction mixture containing 1 μL DNA, 3 μL TaqMan Genotyping Master Mix (Thermo Fisher, Waltham, MA, USA), 0.015 μL each primer, 0.012 μL FAM- and HEX-labelled TaqMan-MGB probes and 1 μL DNA. The program of amplification contained 10 min heat preservation at 95 ${}^{{}^{\circ}}$C followed by 50 cycles of 15 s at 95 ${}^{{}^{\circ}}$C and annealing at 60 ${}^{{}^{\circ}}$C for 1 min. The normalized intensities of the two reporter dyes in each sample were plotted on an allelic discrimination plot and were algorithmically clustered. Genotype calls were assigned according to the sample position on the allelic discrimination plot.

### 2.4. Statistical Analysis

All data were analyzed using SPSS 20.0 statistical software (IBM, Armonk, NY, USA). The χ2-test was used to verify whether the allele frequency of the polymorphism met the Hardy–Weinberg (H-W) equilibrium. Pearson’s chi-square and Mann–Whitney tests were used to compare the demographic and clinical variables between the study groups. Pearson’s Chi-square was used to compare the frequency distribution of genotypes and alleles among groups. The logistic regressions were adjusted for age and gender in this study. A significance level of *p* < 0.05 was considered for all statistical analyses. The associations between different genotypes and lung cancer risk in patients with pulmonary nodule were estimated with an individual odds ratio (OR) and 95% confidence interval (CI).

## 3. Results

### 3.1. Baseline Characteristic of Study Subjects

In the results of the demographic and clinical analyses, it can be observed that the groups differed in terms of age. No significant variation in gender was found between lung cancer patients and benign lung patients (*p* > 0.05). Most cases of lung cancer were LUAD, followed by SCLC and SCC. Among the 400 patients with pulmonary nodules included in the study, a total of 146 patients were tested for EGFR gene mutation. There was no significant difference in EGFR gene mutation between lung cancer patients and benign lung disease patients (*p* = 1.000). It is noteworthy that EGFR mutations exist in patients with benign nodules (Table 1).

### 3.2. Correlations of Allele and Genotype Frequencies of ACE1 rs4646994 Polymorphism with Risk of Lung Cancer in Pulmonary Nodules

The distribution of the ACE1 rs4646994 genotype and allele in the case and control groups is shown in Table 2. The results found that the ID genotype of ACE1 rs4646994 was predominate in both groups. The DD genotype in case group (OR = 2.760, 95% CI, 1.095–6.953, *p* = 0.031) was higher than that in the control group, suggesting that the DD genotype is closely associated with the risk of lung cancer. Compared with the I carrier (II + ID) genotype, the DD genotype significantly increased the onset risk of lung cancer (OR = 3.035, 95% CI, 1.252–7.356, *p* = 0.014). In the female subgroup (Table S1), patients with pulmonary nodules carrying DD genotype have an increased risk of lung cancer (OR = 3.783, 95% CI, 1.057–13.536, *p* = 0.041). Compared with the I carrier (II + ID) genotype, DD genotype (OR = 3.570, 95% CI, 1.036–12.307, *p* = 0.044). The results shown in Table S4 illustrated that DD genotype could increase the risk of lung cancer in older (>45 years) patients with pulmonary nodules (OR = 3.111, 95% CI, 1.119–8.651, *p* = 0.030).

### 3.3. Correlations of Allele and Genotype Frequencies of ACE1 rs4646994 Polymorphism with Histological Types of SCC and LUAD

The distribution of genotypic frequencies of ACE1 rs4646994 in the SCC, LUAD, and control group is listed in Table 3. Patients with pulmonary nodules carrying the DD genotype are about 2.7 times as likely to develop lung adenocarcinoma (OR = 2.756, 95% CI, 1.090–6.968, *p* = 0.032). Compared with the I genotype (II + ID) carriers, patients with the DD genotype are about three times more likely to develop LUAD than patients with benign nodules (OR = 2.963, 95% CI, 1.219–7.199, *p* = 0.016). The comparison of ACE1 rs4646994 between the SCC group and control group manifested no statistical significance (*p* > 0.05). In addition, there is no significant difference between lung squamous cell carcinoma and lung adenocarcinoma in the polymorphism of ACE1 rs4646994 (*p* > 0.05). As shown in Table S2, there was no significant difference between SCC group and LUAD group in the polymorphism of ACE1 rs4646994 by gender stratification analysis (*p* > 0.05). Table S5 showed that there was no correlation between ACE1 rs4646994 gene polymorphism and different histological types of lung cancer at different ages (both *p* > 0.05).

As shown in Table 4, the DD genotype is more frequent in lung adenocarcinoma patients without the EGFR mutation (22.6%). The risk of lung adenocarcinoma in patients with EGFR mutation-positive pulmonary nodules carrying the DD genotype (OR = 0.312, 95% CI, 0.101–0.968, *p* = 0.044) is lower than that in patients carrying genotype II. The DD genotype decreases the onset risk EGFR mutation when compared with the I carrier (II + ID) genotype (OR = 0.304, 95% CI, 0.105–0.875, *p* = 0.027). No evidence shown there was relationship between ACE1 rs4646994 gene polymorphism and EGFR mutation in male (*p* > 0.05) and female (*p* > 0.05) lung adenocarcinoma patients (Table S3). As shown in Table S6, ACE1 rs4646994 gene polymorphism was not found to be associated with EGFR mutation in patients with LUAD by age stratification analysis (*p* > 0.05).

## 4. Discussion

Angiotensin-converting enzyme (ACE) gene polymorphism is one of the most studied genetic systems in recent years, including cardiovascular, metabolic, immune, cancer, aging, neurodegenerative diseases and mental illness [21,22,23,24,25]. This case–control study aimed to investigate the potential association between ACE1 rs4646994 polymorphism and lung cancer risk in Chinese patients with pulmonary nodules, and whether this association is related to lung cancer histology and EGFR mutation. We found that the DD genotype of ACE1 rs4646994 may be associated with lung cancer in patients with pulmonary nodules (OR = 2.760, 95% CI, 1.095–6.953, *p* = 0.031). The results of a previous meta-analysis did not show any significant association between ACE1 rs4646994 polymorphism and lung cancer risk [12]. The inconsistency between the results of this study and our results may be affected by race and the number of samples. We did not observe strong evidence that increased risk was associated with specific histological types of lung cancer (OR = 2.169, 95% CI, 0.239–19.728, *p* = 0.492). More interestingly, we found that patients with pulmonary nodules carrying the DD genotype had an increased risk of adenocarcinoma and a lower probability of EGFR gene mutation (OR = 0.312, 95% CI, 0.101–0.968, *p* = 0.044).

In the gender stratification analysis, we found that DD genotype carriers of ACE1 rs4646994 had a significantly increased risk of lung cancer in female patients with pulmonary nodules (OR = 3.783, 95% CI, 1.057–13.536, *p* = 0.041). No statistically significant association between ACE1 gene polymorphism and lung cancer risk was found in male patients with pulmonary nodules (OR = 1.667, 95% CI, 0.429–6.475, *p* = 0.461). However, it is certain that in both male and female subgroups, we observed that the DD genotype of ACE1 was positively correlated with lung cancer susceptibility in patients with pulmonary nodules. Male patients with pulmonary nodules carrying the DD genotype of ACE1 rs4646994 were more strongly associated with lung adenocarcinoma than lung squamous cell carcinoma (OR = 2.169, 95% CI, 0.239–19.728, *p* = 0.492). Due to the limited number of samples, there was no lung squamous cell carcinoma in female patients diagnosed with lung cancer. There was no significant correlation between the ACE1 polymorphism and EGFR mutation after gender stratification. However, we observed that patients with the DD genotype of ACE1 rs4646994 were less likely to have EGFR mutations in male and female subgroups of lung adenocarcinoma patients, which was consistent with the results of no gender stratification analysis. It is noteworthy that in this study, we observed that patients with benign nodules also had EGFR mutations, suggesting that patients with benign nodules should also be screened for EGFR mutations.

In patients with pulmonary nodules under 45 years old, no statistical association was found between ACE1 rs4646994 and lung cancer risk, while in older patients (>45), the risk of lung cancer was significantly increased in patients with the DD genotype (DD vs. ID + II: OR = 3.423, 95% CI, 1.284–9.126, *p* = 0.014). It was reported that advanced age is a risk factor for lung cancer and pulmonary nodule [26,27,28]. In the age-stratification analysis, no significant correlation was found between ACE1 rs4646994 polymorphism and different pathological types of lung cancer and EGFR mutation in patients with lung adenocarcinoma.

As far as we know, this is the first study exploring the association between ACE1 rs4646994 polymorphism and lung cancer risk in patients with pulmonary nodules. This study is also one of the few that has investigated the association between gene polymorphism and the risk of SCC and LUAD. However, there are some limitations in our research. It is difficult to avoid selection bias and information bias in the whole research process. Although the number of samples included in this study meets the experimental requirements, the number of respondents in the stratified analysis is not sufficient. In order to further understand the role of the ACE1 gene in the development of lung cancer and determine the complex relationship between ACE1 polymorphism and gene–environment interaction, a larger sample size and various expression studies are needed. In addition, as with all association studies, it is certainly necessary to reproduce our findings in independent studies and with different ethnic populations.

## 5. Conclusions

In conclusion, our study indicates that the DD genotype of ACE1 rs4646994 may contribute to an increased risk of lung cancer in patients with pulmonary nodules. Furthermore, the possibility of EGFR mutation in lung adenocarcinoma patients with the ACE1 rs4646994 DD genotype was lower than that of II or ID genotype carriers.

## Figures and Tables

**Table 1:** Demographic and clinical characteristics of the investigated groups.

Characteristics	Case (n = 300)	Control (n = 100)	*p*-Value
Gender, n (%)			0.862 ^a^
Male	135 (45%)	44 (44%)	
Female	165 (55%)	56 (56%)	
Age (years)			
Median (p25–p75%)	57.00 (49.00–64.00)	55.00 (48.25–61.00)	0.038 ^b^*
Histology			
Squamous Cell Carcinoma	14 (5.3%)	-	
Adencarcinoma	282 (93.4%)	-	NA
Small Cell Carcinoma	2 (0.6%)	-	
Malt	2 (0.6%)	-	
EGFR Mutation-positive			1.000 ^c^
Yes	75 (52.1%)	1 (50%)	
No	69 (47.9%)	1 (50%)	

NA, not applicable; -, no data. ^a^ Chi-square test; ^b^ Mann–Whitney; ^c^ Chi-square test with Yates’ continuity correction. * *p*-value < 0.05.

**Table 2:** Genotypes and allele frequencies of ACE1 rs4646994 polymorphisms in patients with lung cancer and benign pulmonary nodules.

Genotype/Allele	Case (n = 300)	Control (n = 100)	*p*-Value ^a^	OR	95% CI
II	121	41		Reference	
ID	132	53	0.474	0.839	0.519–1.356
DD	47	6	0.031	2.760	1.095–6.953
DD vs. ID + II			0.014	3.035	1.252–7.356
I allele	374	135		Reference	
D allele	226	65	0.167	1.271	0.904–1.786

CI, confidence interval; OR, odds ratio. ^a^ Logistic regression adjusted for sex and age. Case group versus control group.

**Table 3:** Correlations of genotype polymorphism in ACE1 rs4646994 with histological types of lung squamous carcinoma and adenocarcinoma.

Genotype/	SCC	LUAD	Control	*p*-Value ^a†^	OR ^†^	95%CI ^†^	*p*-Value ^a‡^	OR ^‡^	95%CI ^‡^	*p*-Value ^a§^	OR ^§^	95%CI ^§^
Allele	(n = 14)	(n = 282)	(n = 100)									
II	7 (50%)	112 (39.7%)	41 (41%)		Reference			Reference			Reference	
ID	6 (42.9%)	126 (44.7%)	53 (53%)	0.153	0.354	0.085–1.469	0.593	0.876	0.540–1.422	0.363	1.751	0.524–5.857
DD	1 (7.1%)	44 (14.6%)	6 (6%)	0.499	0.367	0.020–6.708	0.032	2.756	1.090–6.968	0.492	2.169	0.239–19.728
DD vs. ID + II				0.783	0.679	0.043–10.709	0.016	2.963	1.219–7.199	0.647	1.649	0.194–14.040
I allele	20 (71.4%)	350 (62.1%)	135		Reference			Reference			Reference	
D allele	8 (28.6%)	214 (37.9%)	65	0.696	0.845	0.362–1.972	0.139	1.279	0.919–1.821	0.330	1.551	0.642–3.748

CI, confidence interval; OR, odds ratio. ^†^ SCC group versus control group; ^‡^ LUAD group versus control group; ^§^ LUAD group versus SCC group. ^a^ Logistic regression adjusted for sex and age.

**Table 4:** Correlations of ACE1 rs4646994 genotype polymorphism with EGFR mutation in lung adenocarcinoma.

Genotype/Allele	EGFR+ (n = 75)	EGFR (n = 62)	*p*-Value ^a^	OR	95% CI
II	32 (42.7%)	22 (35.5%)		Reference	
ID	37 (49.3%)	26 (41.9%)	0.894	1.054	0.490–2.264
DD	6 (8.0%)	14 (22.6%)	0.044	0.312	0.101–0.968
DD vs. ID + II	69 (57.3%)	48 (64.5%)	0.027	0.304	0.105–0.875
I allele	101 (67.3%)	70 (56.5%)		Reference	
D allele	49 (32.7%)	54 (43.5%)	0.065	0.628	0.383–1.029

CI, confidence interval; OR, odds ratio. Case group versus control group. ^a^ Logistic regression adjusted for sex and age.

## Data Availability

The datasets employed to support this study are available from the corresponding author upon reasonable request.

## Supplementary Materials

The following supporting information can be downloaded at https://www.mdpi.com/article/10.3390/biomedicines11061549/s1. Table S1. Correlations of genotype polymorphism in ACE1 rs4646994 and lung cancer risk in patients with pulmonary nodules according to gender; Table S2. Correlations of genotype polymorphism in ACE1 rs4646994 with histological types of SCC and adenocarcinoma according to gender; Table S3. Correlations of ACE1 rs4646994 genotype polymorphism with EGFR mutation in lung adenocarcinoma according to gender; Table S4. Correlations of genotype polymorphism in ACE1 rs4646994 and lung cancer risk in patients with pulmonary nodules according to age; Table S5. Correlations of genotype polymorphism in ACE1 rs4646994 with histological types of SCC and adenocarcinoma according to age; Table S6. Correlations of ACE1 rs4646994 genotype polymorphism with EGFR mutation in lung adenocarcinoma according to age.

## Abbreviations

ACE	angiotensin-converting enzyme
CI	confidence intervals
LUAD	adenocarcinoma
NSCLC	non-small-cell lung cancer
OR	odds ratios
SCC	squamous-cell carcinoma
SCLC	small-cell lung cancer
SNPs	single-nucleotide polymorphisms
