# Supplementary material for: Genetic Polymorphisms of ACE1 Rs4646994 Associated with Lung Cancer in Patients with Pulmonary Nodules: A Case–Control Study
Source: Biomedicines. 2023 May 26;11(6):1549. doi: 10.3390/biomedicines11061549 (PMC10294966; doi:10.3390/biomedicines11061549)
Supplement: Supplementary file 1 [file biomedicines-11-01549-s001.zip › biomedicines-2382560-supplementary.pdf]

*Supplementary meterials*

# Genetic Polymorphisms of ACE1 Rs4646994 Associated with Lung Cancer in Patients with Pulmonary Nodules: A Case–Control Study

Rong Qiao[1,†], Siyao Sang[2,†], Jiajun Teng[1,†], Hua Zhong[1,*] , Hui Li[2,3,*], Baohui Han[1,*]

**Table S1.** Correlations of genotype polymorphism in ACE1 rs4646994 and lung cancer risk in patients with pulmonary nodules according to gender.

| Gender | Genotype /allele | Case (n=300) | Control (n=100) | *p*-Value [a] | OR | 95%CI |
|---|---|---|---|---|---|---|
| **Male** | | 135 | 44 | | | |
| | II | 52 (38.5%) | 13 (29.5%) | Reference | | |
| | ID | 63 (46.7%) | 28 (63.6%) | 0.132 | 0.559 | 0.262-1.191 |
| | DD | 20 (14.8%) | 3 (6.8%) | 0.461 | 1.667 | 0.429-6.475 |
| | DD vs. ID+II | | | 0.157 | 2.502 | 0.702-8.916 |
| | I allele | 167 (61.9%) | 54 (61.4%) | Reference | | |
| | D allele | 103 (38.1%) | 34 (38.6%) | 0.978 | 0.993 | 0.604-1.632 |
| **Female** | | 165 | 56 | | | |
| | II | 69 (41.8%) | 28 (50%) | Reference | | |
| | ID | 69 (41.8%) | 25 (44.6%) | 0.715 | 1.126 | 0.596-2.127 |
| | DD | 27 (16.4%) | 3 (5.4%) | 0.041 | 3.783 | 1.057-13.536 |
| | DD vs. ID+II | | | 0.044 | 3.570 | 1.036-12.307 |
| | I allele | 207 (62.7%) | 81 (72.3%) | Reference | | |
| | D allele | 123 (37.3%) | 31 (27.7%) | 0.059 | 1.574 | 0.982-2.523 |

CI, confidence interval; OR, odds ratio. [a] Logistic regression adjusted for age.
Case group versus control group.

**Table S2.** Correlations of genotype polymorphism in ACE1 rs4646994 with histological types of SCC and adenocarcinoma according to gender.

| Gender | Genotype /allele | LUAD (n=282) | SCC (n=14) | *p*-Value [a] | OR | 95%CI |
|---|---|---|---|---|---|---|
| **Male** | | 118 | 14 | | | |
| | II | 43 (36.4%) | 7 (50%) | Reference | | |
| | ID | 57 (48.3%) | 6 (42.9%) | 0.363 | 1.751 | 0.524-5.857 |
| | DD | 18 (15.3%) | 1 (7.1%) | 0.492 | 2.169 | 0.239-19.728 |
| | DD vs. ID+II | | | 0.426 | 2.340 | 0.288-19.012 |
| | I allele | 143 (60.6%) | 20 (71.4%) | reference | | |
| | D allele | 93 (39.4%) | 8 (28.6%) | 0.647 | 1.649 | 0.194-14.040 |

| | | | | | | |
|---|---|---|---|---|---|---|
| **Female** | | 164 | 0 | | | |
| | II | 69 (42.1%) | 0 | Reference | | |
| | ID | 69 (42.1%) | 0 | - | - | - |
| | DD | 26 (15.9) | 0 | - | - | - |
| | DD vs. ID+II | | | - | - | - |
| | I allele | 207 (63.1%) | 0 | Reference | | |
| | D allele | 121 (36.9%) | 0 | - | - | - |

CI, confidence interval; OR, odds ratio. [a] Logistic regression adjusted for age.
LUAD group versus SCC group.

**Table S3.** Correlations of ACE1 rs4646994 genotype polymorphism with EGFR mutation in lung adenocarcinoma according to gender.

| **Gender** | **Genotype /allele** | **EGFR+ (n=75)** | **EGFR (n=62)** | ***p*-Value [a]** | **OR** | **95%CI** |
|---|---|---|---|---|---|---|
| **Male** | | 29 | 32 | | | |
| | II | 10 (34.5%) | 11 (34.4%) | Reference | | |
| | ID | 18 (62.1%) | 14 (43.8%) | 0.612 | 1.335 | 0.436-4.086 |
| | DD | 1 (3.4%) | 7 (21.9%) | 0.129 | 0.171 | 0.018-1.670 |
| | DD vs. ID+II | | | 0.083 | 0.145 | 0.016-1.284 |
| | I allele | 38 (65.5%) | 36 (56.2%) | Reference | | |
| | D allele | 20 (34.5%) | 28 (43.8%) | 0.253 | 0.662 | 0.325-1.344 |
| **Female** | | 46 | 30 | | | |
| | II | 22 (47.8%) | 11 (36.7%) | Reference | | |
| | ID | 19 (41.3%) | 12 (40%) | 0.759 | 0.848 | 0.296-2.409 |
| | DD | 5 (10.9%) | 7 (23.3z%) | 0.176 | 0.384 | 0.096-1.534 |
| | DD vs. ID+II | | | 0.180 | 0.416 | 0.115-1.501 |
| | I allele | 63 (68.5%) | 34 (56.7%) | Reference | | |
| | D allele | 29 (31.5%) | 26 (43.3%) | 0.190 | 0.630 | 0.315-1.257 |

CI, confidence interval; OR, odds ratio. [a] Logistic regression adjusted for age.
EGFR+ group versus EGFR group.

**Table S4.** Correlations of genotype polymorphism in ACE1 rs4646994 and lung cancer risk in patients with pulmonary nodules according to age.

| **Age** | **Genotype /allele** | **Case (n=300)** | **Control (n=100)** | ***p*-Value [a]** | **OR** | **95%CI** |
|---|---|---|---|---|---|---|
| **≤45** | | 48 | 17 | | | |
| | II | 20 (41.7%) | 7 (41.2%) | Reference | | |
| | ID | 21 (43.8) | 9 (52.9%) | 0.669 | 0.767 | 0.227-2.590 |
| | DD | 7 (14.6) | 1 (5.9%) | 0.498 | 2.216 | 0.222-22.154 |
| | DD vs. ID+II | | | 0.399 | 2.573 | 0.287-23.077 |
| | I allele | 201 (63.8%) | 50 (61.0%) | Reference | | |
| | D allele | 114 (36.2%) | 32 (39.0%) | 0.625 | 0.882 | 0.534-1.458 |
| **>45** | | 252 | 83 | | | |
| | II | 101 (40.1%) | 34 (41.0%) | Reference | | |

| | | | | | | |
|---|---|---|---|---|---|---|
| | ID | 111 (44.0%) | 44 (53.0%) | 0.512 | 0.838 | 0.493-1.423 |
| | DD | 40 (15.9%) | 5 (6%) | 0.030 | 3.111 | 1.119-8.651 |
| | DD vs. ID+II | | | 0.014 | 3.423 | 1.284-9.126 |
| | I allele | 313 (62.1%) | 112 (67.5%) | Reference | | |
| | D allele | 191 (37.9%) | 54 (32.5%) | 0.146 | 1.322 | 0.908-1.926 |

CI, confidence interval; OR, odds ratio. [a] Logistic regression adjusted for sex and age.
Case group versus control group.

**Table S5.** Correlations of genotype polymorphism in ACE1 rs4646994 with histological types of SCC and adenocarcinoma according to age.

| **Age** | **Genotype /allele** | **LUAD (n=282)** | **SCC (n=14)** | ***p*-Value [a]** | **OR** | **95%CI** |
|---|---|---|---|---|---|---|
| **≤45** | | 48 | 0 | | | |
| | II | 20 (41.7%) | 0 | | Reference | |
| | ID | 21 (43.8%) | 0 | - | - | - |
| | DD | 7 (14.6%) | 0 | - | - | - |
| | DD vs. ID+II | - | - | - | - | - |
| | I allele | 61 (63.5%) | 0 | | Reference | |
| | D allele | 35 (36.5%) | 0 | - | - | - |
| **>45** | | 234 | 14 | | | |
| | II | 92 (39.3%) | 7 (50%) | | Reference | |
| | ID | 105 (44.9%) | 6 (42.9%) | 0.458 | 1.556 | 0.484-4.996 |
| | DD | 37 (15.8%) | 1 (7.1%) | 0.342 | 2.872 | 0.326-25.268 |
| | DD vs. ID+II | | | 0.441 | 2.286 | 0.279-18.707 |
| | I allele | 289 (61.8%) | 20 (71.4%) | Reference | | |
| | D allele | 179 (38.2%) | 8 (28.6%) | 0.277 | 1.615 | 0.680-3.835 |

CI, confidence interval; OR, odds ratio. [a] Logistic regression adjusted for sex.
LUAD group versus SCC group.

**Table S6.** Correlations of ACE1 rs4646994 genotype polymorphism with EGFR mutation in lung adenocarcinoma according to age.

| **Age** | **Genotype /allele** | **EGFR+ (n=75)** | **EGFR (n=62)** | ***p*-Value [a]** | **OR** | **95%CI** |
|---|---|---|---|---|---|---|
| **≤45** | | 9 | 10 | | | |
| | II | 4 (44.4%) | 3 (30%) | | Reference | |
| | ID | 5 (55.6%) | 4 (40%) | 0.942 | 0.929 | 0.126-6.842 |
| | DD | 0 | 3 (30%) | 0.999 | 0 | 0 |
| | DD vs. ID+II | | | 0.999 | 0 | 0 |
| | I allele | 13 (72.2%) | 10 (50%) | | Reference | |
| | D allele | 5 (27.8%) | 10 (50%) | 0.183 | 0.396 | 0.102-1.548 |
| **>45** | | 66 | 52 | | | |
| | II | 28 (42.4%) | 19 (36.5%) | | Reference | |
| | ID | 32 (48.5%) | 22 (42.3%) | 0.824 | 1.097 | 0.485-2.481 |
| | DD | 6 (9.1%) | 11 (21.2%) | 0.084 | 0.356 | 0.110-1.148 |

| | | | | | |
|---|---|---|---|---|---|
| DD vs. ID+II | | | 0.053 | 0.340 | 0.114-1.013 |
| I allele | 88 (66.7%) | 60 (57.7%) | | Reference | |
| D allele | 44 (33.3%) | 44 (42.3%) | 0.175 | 1.451 | 0.848-2.484 |

CI, confidence interval; OR, odds ratio. [a] Logistic regression adjusted for sex.

EGFR+ group versus EGFR group.
